# Effectiveness of a Machine Learning-Enabled Skincare Recommendation for Mild-to-Moderate Acne Vulgaris: 8-Week Evaluator-Blinded Randomized Controlled Trial

**DOI:** 10.2196/60883

**Published:** 2025-07-16

**Authors:** Misbah Noshela Ghazanfar, Ali Al-Mousawi, Christian Riemer, Benóný Þór Björnsson, Charlotte Boissard, Ivy Lee, Zarqa Ali, Simon Francis Thomsen

**Affiliations:** 1 Department of Dermatology, Bispebjerg Hospital, Bispebjerg bakke 23, Copenhagen, 2400, Denmark; 2 NØIE, Copenhagen, Denmark; 3 Division of Dermatology, University of California Los Angeles, Los Angeles, CA, United States; 4 Department of Biomedical Sciences, University of Copenhagen, Copenhagen, Denmark

**Keywords:** machine learning, personalised skincare, acne vulgaris, dermatology, skincare

## Abstract

**Background:**

Acne vulgaris (AV) is one of the most common skin disorders, with a peak incidence in adolescence and early adulthood. Topical treatments are usually used for mild to moderate AV; however, a lack of adherence to topical treatment is seen in patients due to various reasons. Therefore, personalized skincare recommendations may be beneficial for treating mild-to-moderate AV.

**Objective:**

This study aimed to evaluate the effectiveness of a novel machine learning approach in predicting the optimal treatment for mild-to-moderate AV based on self-assessment and objective measures.

**Methods:**

A randomized, evaluator-blinded, parallel-group study was conducted on 100 patients recruited from an internet-based database and randomized in a 1:1 ratio (groups A and B) based on their consent form submission. Groups A and B received customized product recommendations using a Bayesian machine learning model and self-selected treatments, respectively. The patients submitted self-assessed disease scores and photographs after the 8-week treatment. The primary and secondary outcomes were photograph evaluation by two board-certified dermatologists using the Investigator Global Assessment (IGA) scores and quality of life (QoL) measured using the Dermatology Life Quality Index (DLQI), respectively.

**Results:**

Overall, 99 patients were screened, and 68 patients (mean age: 27 years, SD 4.56 years) were randomized into groups A (customized) and B (self-selected). IGA scores significantly improved after treatment in group A but not in group B (mean difference in IGA score; group A=0.32, *P*=.04 vs group B=0.09, *P*=.54). The DLQI significantly improved in group A from 7.75 at baseline to 3.5 (*P*<.001) after treatment but reduced in group B from 7.53 to 5.3 (*P*>.05). IGA scores and the DLQI were significantly correlated in group A, but not in group B. A total of 3 patients reported adverse reactions in group B, but none in group A.

**Conclusions:**

Using a machine learning model for personalized skincare recommendations significantly reduced symptoms and improved severity and overall QoL of patients with mild-to-moderate AV, supporting the potential of machine learning-based personalized treatment options in dermatology.

## Introduction

Acne vulgaris (AV) is one of the most common skin disorders, with a peak incidence in adolescence and early adulthood, affecting approximately 85% of individuals aged 12‐24 years. Although acne is most prevalent in teenagers, it can emerge at any age. Approximately 25% and 12% of women and men in their 40s, respectively, report experiencing acne, usually accompanied by a high degree of stigma and impairment in quality of life (QoL) [1].

Topical treatments, such as retinoids, antibiotics, and combinations of antibiotics and benzoyl peroxide, are usually used for mild-to-moderate AV [2]; however, there is a lack of adherence to topical treatments among patients with AV [3]. A wide range of skincare products are available at beauty stores, pharmacies, and web-based shops. Many of these products are inefficient, and users usually lack knowledge about which ingredients are effective and beneficial for their skin condition.

A Danish skincare company (NØIE) has developed a method for customizing skincare products based on in-depth phenotyping and direct feedback loops from over 80,000 patients with a skin condition by collecting clinical data on skin characteristics during an web-based survey and combining it with dermatological knowledge, feedback from users, and statistical modeling. In 2019, after 3 years of development, the project successfully launched a data model that modeled personalized skincare solutions based on an individual’s specific skin and personal needs.

Therefore, this study aimed to evaluate the effectiveness of a novel machine learning approach for predicting the optimal treatment for mild-to-moderate AV based on subjective patient self-assessment and objective measures, including the physician-rated Investigator Global Assessment (IGA) and the patient-rated Dermatology Life Quality Index (DLQI).

## Methods

### Study Design and Participants

This evaluator-blinded, randomized controlled parallel-group trial included 100 patients who were randomized into groups A and B on a 1:1 ratio based on their submission of consent forms. It was conducted in accordance with the CONSORT (Consolidated Standards of Reporting Trials) statement (Checklist 1) [4]. Each group was assessed and assigned treatment based on the self-assessed reporting and image or real-life face-to-face interaction: group A used NØIE’s Bayesian machine learning model and group B found and chose products themselves. The patients submitted self-assessed disease scores and a standardized set of facial images, known as a collected dataset, as the skin profile after 8 weeks of adherence to treatment. Subsequently, these assessments and images were scored by 2 independent board-certified dermatologists to evaluate the effectiveness of the 2 methods.

### Recruitment Process

Patients were recruited through several channels affiliated with NØIE and e-mail campaigns targeting NØIE’s database. NØIE contacted respondents to determine eligibility via a short survey before the screening process. The eligibility criteria were patients aged 18‐40 years with a known diagnosis of AV who were interested in participating in the study from mid-February onwards for an 8-week duration and resided in Denmark, Germany, the Netherlands, Sweden, the United Kingdom, Switzerland, Belgium, or Austria.

All patients were required to submit a high-resolution image of their facial acne, which an employee of NØIE objectively assessed to confirm the AV and ensure that the disease severity was mild-to-moderate based on lesion counts. Mild AV was categorized as mostly whiteheads and blackheads with a few papules and pustules, whereas moderate acne was categorized as multiple papules and pustules.

The inclusion criteria were healthy female or male patients aged 18‐40 years with mild-to-moderate AV and who had not previously used NØIE products. In contrast, the exclusion criteria were pregnancy, breastfeeding, or any changes in birth control during the intervention period since these would cause fluctuations in the hormonal impact on disease severity. Patients who used prescription medical treatments for acne treatment were also excluded.

### Bayesian Model Guidance for Product Development

NØIE is established upon substantial data collection from over 65,000 individuals with various skin diseases, including skin reactions to well-categorized products commercially sold across Europe. These data were reverse-engineered into a matrix of stratified user segmentation, with an underlying layer of products developed by NØIE to support the optimal needs of individual user’s skin. In addition, this precision medicine approach has only been used in oncology to date; however, NØIE has incorporated Bayesian modeling to stratify users based on differences in their epigenetic features, lifestyle, and personal preferences and their response to the products being used, based on the collected data, in-house product development, and direct contact with each user. The feedback from real-world data not only trains the model for precision but also guides skincare product development simultaneously to better meet the diverse needs of patients.

Precision medicine is an emerging approach in clinical research and patient care that focuses on understanding and treating diseases by integrating multimodal or multiomics data from each patient to make personalized treatment decisions [5]. In addition, dealing with the large and intricate datasets generated by precision medicine diagnostics requires the development of innovative techniques to process and interpret this complex information. Concurrently, rapid advancements in computer science have enabled the storage, processing, and analysis of these intricate datasets—a task that traditional statistics and early computing technologies could not accomplish. Therefore, the Bayesian modeling approach provides a means to formalize previous beliefs and combine them with available observations, aiming to derive rational criteria for optimal decision-making and measure the outcomes of these decisions [5].

This approach forms the foundational core of NØIE, aiming to identify intricate patterns in data for making predictions and classifications and conducting advanced exploratory data analysis on new, unseen data to guide their product development and distribution for better and safer treatments for various phenotypes of a given skin disease.

The fundamental input for the Bayesian model was the patient undergoing a skin test, which is a cumbersome in-depth web-based survey capturing 31 parameters relevant to the underlying skin condition or disease. These different data points are stored in real-time, forming the patient’s skin profile, which the Bayesian models activate to recommend the ideal skin products for providing alleviation. Since all patients are asked to provide feedback on the effectiveness of their given treatment, it establishes a closed loop in the modeling process where results are automatically considered, thereby continuously strengthening the model’s precision.

### Product Description

Since NØIE’s products are classified as cosmetics and medical device class I, the ingredients distinguish themselves from classic active pharmaceutical ingredients known in the pharmaceutical industry, although they rely on synergistic effects between conventionally used cosmetic ingredients and innovative modified peptides. Conventional ingredients include salicylic acid, retinol, and niacinamide, whereas innovative ingredients include *Curcuma longa* callus lysate, *Morinda citrifolia* callus culture, and Lactiplantibacillus fermentation lysate. Both groups’ routine skincare regimens comprised a face cleanser and cream.

### Group A: Customized Skincare

All patients in this group underwent a skin test to create a skin profile. Using the Bayesian model, they received product recommendations including a personalized face cleanser and cream. These products were shipped by NØIE.

### Group B: Self-Selected Skincare

Similar to group A, patients in group B filled out using the same skin profile as their starting point. However, compared with group A, it did not activate NØIE’s machine learning endpoint for a recommendation. All patients in this group were instructed to select a face cream and cleanser that they believed would improve their acne symptoms over 8 weeks. The choice of product was completely at the patients’ discretion; they were permitted to seek advice from family, friends, pharmacists, and doctors but were not allowed medical treatment. NØIE purchased and shipped the self-selected products to patients, or patients purchased the products themselves and NØIE refunded the invoice.

### Adverse Reactions

Patients were instructed to contact NØIE immediately in the event of any adverse reactions. In such cases, patients were given the option to either substitute the product or were automatically assigned an alternative formulation by NØIE’s underlying machine learning model.

### Dermatological Assessment

**Figure 1.**
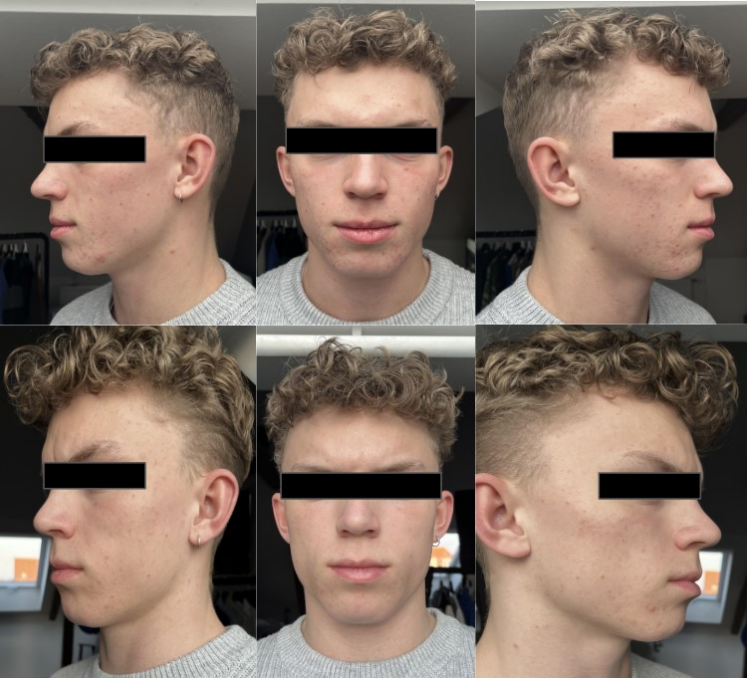
Patient photographs for assessment by dermatologists.

A dermatological assessment was conducted based on three images provided by the patients on days 0 and 56. The 3 images included each cheek side and a frontal profile captured in high resolution, with balanced lighting and without makeup (Figure 1).

The two assessors were licensed dermatologists located in Denmark and the United States of America, respectively, and unaffiliated with NØIE in any sense. In addition, the images were rated according to the IGA [6], and the assessors were blinded to each other’s ratings and the group origin of the assessed participant alongside the three images on days 0 and 56. Therefore, biasing the outcomes of the assessments was impossible.

The triplets of before-and-after images from each participant receiving personalized intervention were randomized so that the same participant’s datasets were not evaluated consecutively. In addition, the order of the images (before and after) was randomized. This ensured that the dermatologist assessed each triplet without bias, as if conducting individual consultations, rather than being influenced by prior comparisons.

After the complete assessment of 72 image triplets, the results were organized in the correct before-and-after sequence for each participant. This allowed for the calculation of the intervention’s development and effectiveness for each individual.

### Primary and Secondary Outcomes

The primary outcome measure was the evaluation of changes in acne severity based on IGA scores by blinded assessors, whereas the secondary outcome was QoL measured using the DLQI. IGA scores were graded from 0 to 4 (from clear 0 to severe acne 4), and only facial acne was included in the assessment. Chest, back, and shoulder acne was not considered in this study. Both inflammatory and noninflammatory lesion counts have been reported [7]. Furthermore, the DLQI is a 10-item retrospective questionnaire that assesses the QoL of patients with skin conditions. Each question has scores ranging from 0 to 3, with a maximum total score of 30. A higher score indicates a greater impairment in QoL, and the minimal clinically important difference for DLQI is 3.10 points [8].

### Self-Assessment

The self-assessment conducted during the study covered five lesion types for the patients. In addition, the question “Do you deal with symptom X*”* comprises a response scale ranging from “Not at all*”* to “Extremely*”*. Subsequently, the positioning on the sliding scale was automatically converted to a numerical grade in NØIE’s backend, where direct “Not at all” and “Extremely*”* are equivalent to 0 and 10, respectively. This implies that patients could score their acne severity from 0 to 50 across all five lesion types. The five acne lesion types assessed were papules, pimples, blackheads, whiteheads, and oiliness, excluding nodules or cysts as they mainly belong to the severe state of AV.

### Changes in Hypothetical Medical Intervention From Blinded Dermatologists

A third-party unaffiliated dermatologist with decades of experience in treating AV across all severities was randomly assigned to the group receiving customized treatment (group A) according to the sequence and the order of the intervention. Based on the 3 photographs, we assessed which treatment to prescribe, if any, as a hypothetical action which could be considered a development caused by the study intervention, as all other parameters remained unchanged. Multimedia Appendix 1 presents the 6 interventions performed by a blinded dermatologist.

Intervention severity was scored from 1 (least severe) to 6 (most severe). The mean severity was calculated at baseline and after the 8-week intervention, as well as the mean difference for each patient. The higher the mean score, the more severe the intervention required. The difference was calculated as the intervention score after the 8-week treatment minus the intervention score at baseline, where a negative score reflects a downgrade in the severity of the intervention required.

A *t* test was performed to determine the difference between the interventions at baseline and after 8 weeks. Statistical significance was set at an α value of .05.

### Statistical Analysis

Differences in mean values within and between groups were analyzed using the *t* test where normalcy and continuity of the samples have been verified and with the nonparametric Mann-Whiteny *U* test where those requirements were not met, along with comparing percentage differences. Correlations were calculated using the Pearson correlation coefficient where applicable, and using Spearman correlation where the normalcy requirement was not met for the Pearson correlation. Statistical analysis, correlation calculations, and graphing were performed using Python version 3.7.12, Pandas library version 1.3.5, Numpy library version 1.21.6, Seaborn library version 0.12.1, and Scipy library version1.11.2.

### Cohen Test to Ensure Adequate Sample Sizes

A power calculation using the Cohen test was conducted before initiating the recruitment process to estimate the sample size required for each group for determining the likelihood of detecting an effect in the experiment if it truly existed. First, the effect size (ES) was calculated by substituting the proportions of patients expected to be improved by each treatment, p_1_=0.86 and p_2_=0.50 (p_1_=0.86 refers to the product satisfaction rate obtained by NØIE within the last 2 years; p_2_=0.5 was set as a high estimate for the self-selected group since competent guidance was expected), and the overall proportion, *P*=.68 (ie, 0.86 [0.50] / 2):


$$ES=\frac{\left|p_{1}-p_{2}\right|}{\sqrt{p\left(1-p\right)}}=\frac{\left|0.86-0.5\right|}{\sqrt{0.68\left(1-0.68\right)}}=0.7717$$

Subsequently, we calculated the sample size (n_i_) for each group (i=1, 2). ES was used in the equation for two independent samples, along with the confidence level, ensuring that a two-sided test with a 5% level of significance (ie, *α*=.05) and 80% power to detect the estimated response difference between the 2 groups. The equation is presented as follows:


$$n_{i}=2{\left(\frac{Z_{1}-\frac{\alpha }{2}+Z_{1}-\beta }{ES}\right)}^{2}=2{\left(\frac{1.96+0.84}{0.7717}\right)}^{2}=26.32$$

The use of n_1_=26 and n_2_=26 ensures that the test of the hypothesis has 80% power to detect a product effect. We anticipated patient dropouts due to strict inclusion criteria and noncompliance with product usage. Therefore, we recruited 50 patients in each group (n_1_=50 and n_2_=50).

### Ethical Considerations

All patients provided written informed consent before enrollment. Approval from the scientific ethics committee was not required as no medical interventions were conducted in the study.

This study investigates the efficacy of skincare products through secondary analysis of consumer data. It is important to note that no medical interventions or treatments were involved in the study. The data used were collected for nonmedical purposes, and all participants had provided informed written consent for their identifiable data to be shared with researchers.

Ethical approval is not needed for this type of study in Denmark, where the intervention is a behavioral change without a medicinal product involved. This study did not involve any clinical treatments, procedures, or health interventions, and since the analysis was based on existing consumer data with appropriate safeguards in place, the study did not meet the criteria for requiring ethical approval from a health research ethics committee, in accordance with the Danish National Committee on Health Research Ethics (NVK) guidelines [6]. The NVK generally requires ethical approval for research involving medical interventions or when sensitive health data is being processed [6].

Furthermore, the data used for publication have been fully anonymized, ensuring the privacy and confidentiality of the participants. The General Data Protection Regulation (GDPR) (Regulation (EU) 2016/679) [9] and the Danish Data Protection Act [10] govern the use of personal data in research and allow for the processing of personal data when explicit consent is obtained and anonymization techniques are applied to protect privacy [10].

Participant enrollment alongside the collected data during the study interventions relied on secondary analysis conducted under a voluntary, waived consent framework. Accordingly, the consent form for study participation included detailed information about both primary data collection and the secondary analysis of the collected data, ensuring comprehensive informed consent for participants regarding the dual-purpose use of their data. The study participants were informed in writing about the study and consented to participate and with an option to contact NØIE in case of queries.

All the data collected on enrolled participants were stored on a secure, compliant server in an anonymized format, with participant identities protected through a unique ID system. Deciphering individual participants required conversion via a separate platform to maintain confidentiality. Access to this encrypted data was restricted solely to NØIE, and only aggregated data, without identifiers, was shared through a compliant system with external assessors. Access was disabled upon completion of the assessment, and NØIE permanently deleted all collected identifiable data exactly one year after collection, effected in May 2024.

None of the enrolled participants received or were offered financial compensation for their participation in the study. However, all participants were uniformly provided with complimentary products corresponding to their respective cohorts throughout the 8-week intervention period.

The publication does not contain any identifiable information about individual participants; all data are aggregated by cohort and lack identifiable elements, with the exception of images.

The appendix includes images of one study participant, anonymized with a censor bar covering the eyes. An additional signed consent form was obtained from this participant, stating:

I, the undersigned, agree to allow NØIE and a team of unaffiliated doctors to showcase my before and after images obtained during the 8 weeks of study involvement as part of summarising the learnings and insights obtained in the study. The intention is for readers of the publication to assess the image quality, and not intended to address any changes seen during the 8 weeks of intervention.

I, the undersigned understand and hereby authorise and agree to NØIE utilising my photos, for scientific and awareness purposes to support aggregated numbers and results published in a physical dermatological journal as well as its corresponding online version. The material with me will likely be used in an intended publication to showcase results of a novel methodology like the AI driven recommendation carried out by NØIE as part of the study.

All images will be eye censored to hinder identification.

Furthermore, no compensation was offered or given to the participant for the consent to use the images in the publication. This study involved no medical intervention; therefore, approval from the ethics committee was not required. Written consent was obtained from all participants before participation in the study.

## Results

### Randomization and Screening

A total of 99 patients were screened for randomization. The failure to obtain the intended 100 patients was attributed to a strict deadline, given the coordination required for product shipment and the need to minimize the period between the initial screening of AV severity and the first day of the intervention.

**Figure 2.**
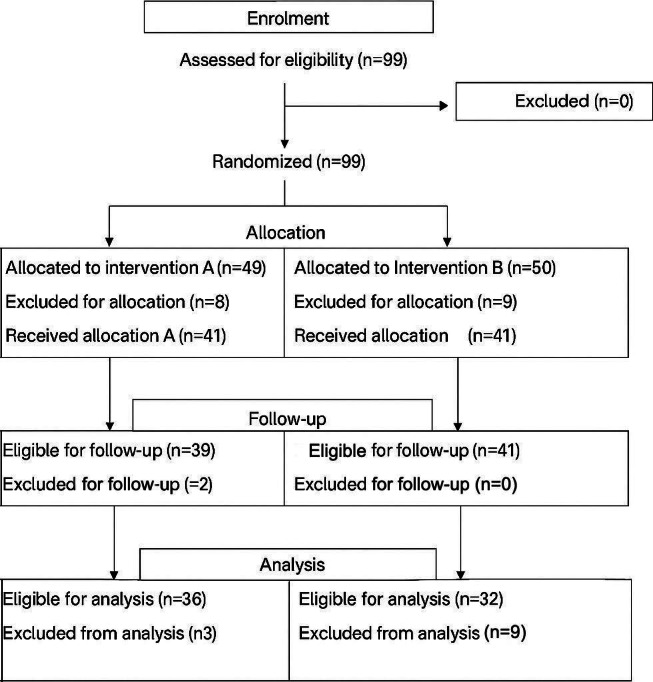
The consolidated standards of reporting trials diagram. IGA: Investigator Global Assessment.

Of the 99 patients, 17 were excluded before randomization because of the inclusion criteria. A total of 5 patients from group A, and 9 patients group B were excluded from data analysis due to noncompliance with usage guidelines (self-reported) or intervention, as shown in Figure 2.

The patients excluded after filling out the initial dataset did not exhibit any specific characteristics regarding age, sex, or disease severity compared with those analyzed in either of the 2 groups. Although modafinil and lamotrigine are not prescribed for AV, they are well-known for their ability to affect the skin and were among the medications causing exclusion due to their potential interference with the study outcome.

In total, 68 patients were randomized into group A (customized), which included 36 patients (34 females and 2 males) with a mean age of 27.1 years, SD 4.76, and group B (self-selected) comprising 32 patients (30 women and 2 men) with a mean age of 26.3 years, SD 4.36 (Table 1). The duration of AV was 5 and 6 years in groups A and B, respectively. In addition, 58.3% and 62.5% of the patients in groups A and B, respectively, had previously undergone medical treatment. Baseline DLQI were 7.75 and 7.53 in groups A and B, respectively, indicating a moderate effect on QoL in both groups.

**Table 1.** Patient characteristics.

Characteristics	Group A (customized)	Group B (self-selected)
Age (years), mean (SD; range)	27.1 (4.76; 20–38)	26.3 (4.36; 19–35)
Sex, n (%)		
Female	34 (94.4)	30 (93.7)
Male	2 (5.5)	2 (6.3)
Duration of AV^a^ (years), mean (SD)	4.94 (3.75)	5.98 (4.84)
Previous use of acne medication, n (%)	19 (58.3)	23 (62.5

a AV: acne vulgaris.

### Primary Outcome: IGA Assessments

The mean (SD) IGA score improved from 1.53 (0.83) at baseline to 1.21 (0.76) after the 8-week treatment in group A (mean difference in IGA score=0.32, *P*=.04) and improved from 1.42 (0.42) at baseline to 1.33 (0.87) after the 8-week treatment in group B (mean difference in IGA score=0.09, *P*=.54) (Table 2).

**Table 2.** Primary and secondary outcomes at baseline and the 8-week follow-up.

Outcomes	Baseline	Follow-up at 8 weeks	*P* value
IGA^a^, mean (SD)			
Group A	1.53 (0.83)	1.21 (0.76)	.03
Group B	1.42 (0.42)	1.33 (0.87)	.53
DLQI^b^, mean (SD)			
Group A	7.75 (5.03)	3.5 (4.1)	<.001
Group B	7.53 (6.16)	5.3 (4.7)	>.05
Self-assessment, mean (SD)			
Group A	26.6 (4.9)	20.3 (7.6)	<.001
Group B	24.9 (7.6)	20.6 (8.44)	.03

a IGA: Investigator Global Assessment.

b DLQI: Dermatology Life Quality Index.

Table 3 illustrates the agreement between the 2 assessors’ IGA scores. The 2 assessors disagreed in 36 cases but agreed upon no change (n=16), improvement (n=13), and regression (n=3) in 32 cases. Significant correlations were observed between the 2 assessors at baseline (*r*=0.452, 95% CI=0.24‐0.62, *P*<.001) and after the 8-week treatment (*r*=0.54, 95% CI 0.35‐0.69; *P*<.001).

**Table 3.** Matrix showing inter-agreements plotted as aggregate numbers for the 2 groups.

Assessor evaluation	Assessor #2 IGA^a^ score reduced	Assessor #2 IGA score remained unchanged	Assessor #2 IGA score increased
Assessor #1 IGA score reduced	13^b^	5^c^	3^d^
Assessor #1 IGA score remained unchanged	9^c^	16^b^	7^c^
Assessor #1 IGA score increased	6^d^	6^c^	3^b^

a IGA: Investigator Global Assessment.

b Consensus between 2 assessors.

c Minor disagreement.

d Contradictory assessments.

### Secondary Outcome: QoL

This parameter measured using the DLQI significantly improved in group A from 7.75 (5.03) at baseline to 3.5 (4.1) after the 8-week treatment (mean difference in DLQI=4.3, *P*<.001). In contrast, a reduction in DLQI from 7.53 (6.16) at baseline to 5.3 (4.7) after the 8-week treatment was observed in group B (mean difference in DLQI=2.3), although this was not significant (*P*>.05; Table 2).

### Correlation Between the IGA Score and the DLQI

**Figure 3.**
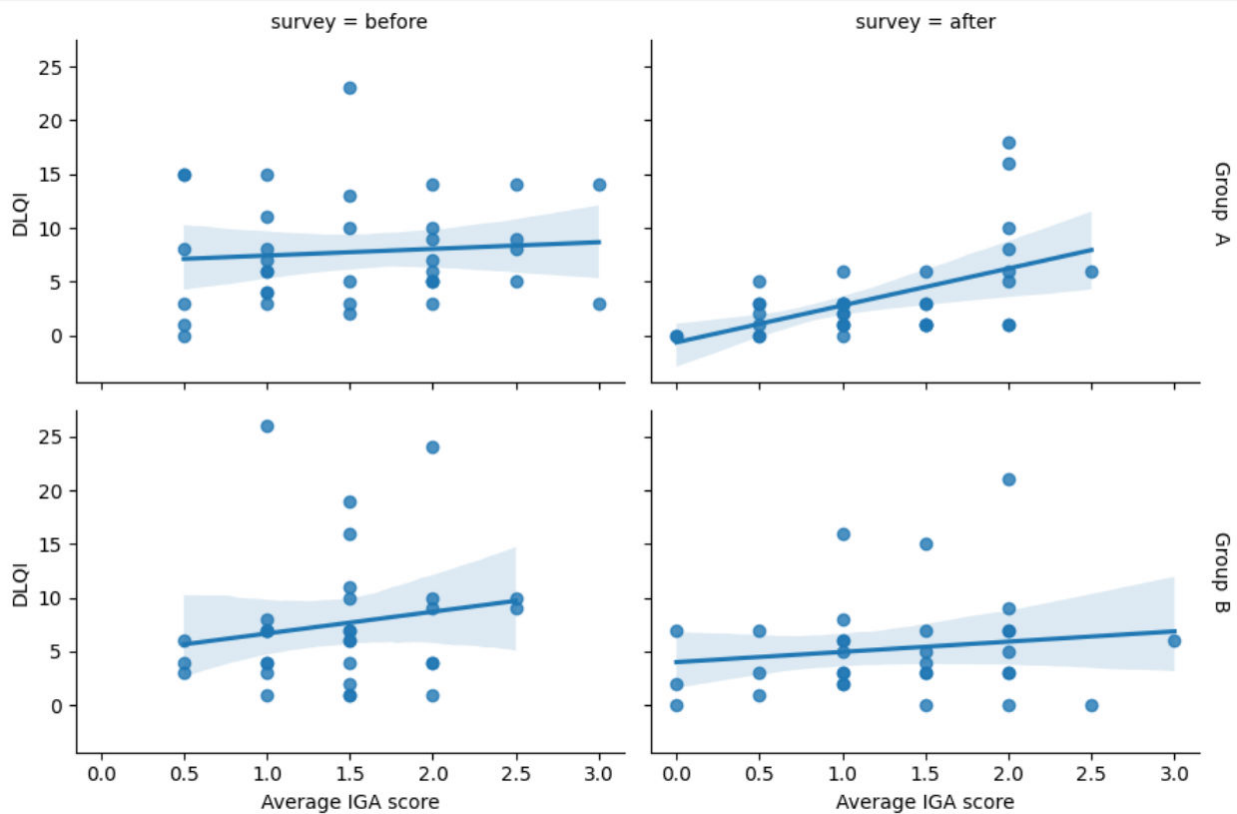
Correlation between acne severity (IGA score) and DLQI before and after intervention in groups A and B. DLQI: Dermatology Life Quality Index; IGA: Investigator Global Assessment.

Weak and moderate correlations were observed between the IGA score and the DLQI at baseline (*r*=0.11, 95% CI −0.23 to 0.42, *P*>.05) and after the 8-week treatment (*r*=0.516, 95% CI 0.23-0.72, *P*<.001) in group A, respectively. However, a weak correlation was observed between acne severity and the DLQI at baseline (*r*=0.242, 95% CI −0.12 to 0.54, *P*>.05) and after the 8-week treatment (*r*=0.136, 95% CI −0.22 to 0.47, *P*>.05) in group B (Figure 3).

### Self-Assessed Symptoms

Group A had a total mean (SD) symptom score of 26.6 (4.9) at baseline, which improved to 20.3 (7.6) after the 8-week treatment (mean difference=6.3, 95% CI 3.22‐9.32, *P*<.001). In contrast, group B had a total mean (SD) symptom score of 24.9 (7.6) at baseline, which improved to 20.6 (8.44) after the 8-week treatment (mean difference=4.31, 95% CI=0.26‐8.37, *P*=.03) (Table 2). Furthermore, significant reductions in the proportions of blackheads (25%, 8/32), whiteheads (31%, 10/32), pimples (26%, 8/32), and skin oiliness (23%, 7/32) were observed in group A after the 8-week follow-up. In total, 61% (20/32) and 56% (20/36) of the patients in groups A and B, respectively, reported that their skin appeared healthier after using the customized skincare routine and the 8-week treatment, respectively. Greater reductions in the proportions of blackheads (53%, 17/32 vs 35%, 13/36) and pimples (67%, 22/32 vs 34%, 12/36) were observed in group A than in group B. Moreover, a significant visual improvement in the skin was observed in group A compared with that in group B (67%, 22/32 vs 37%, 13/36, *P*=.008).

### Correlation Between Self-Assessed Symptoms and the DLQI

**Figure 4.**
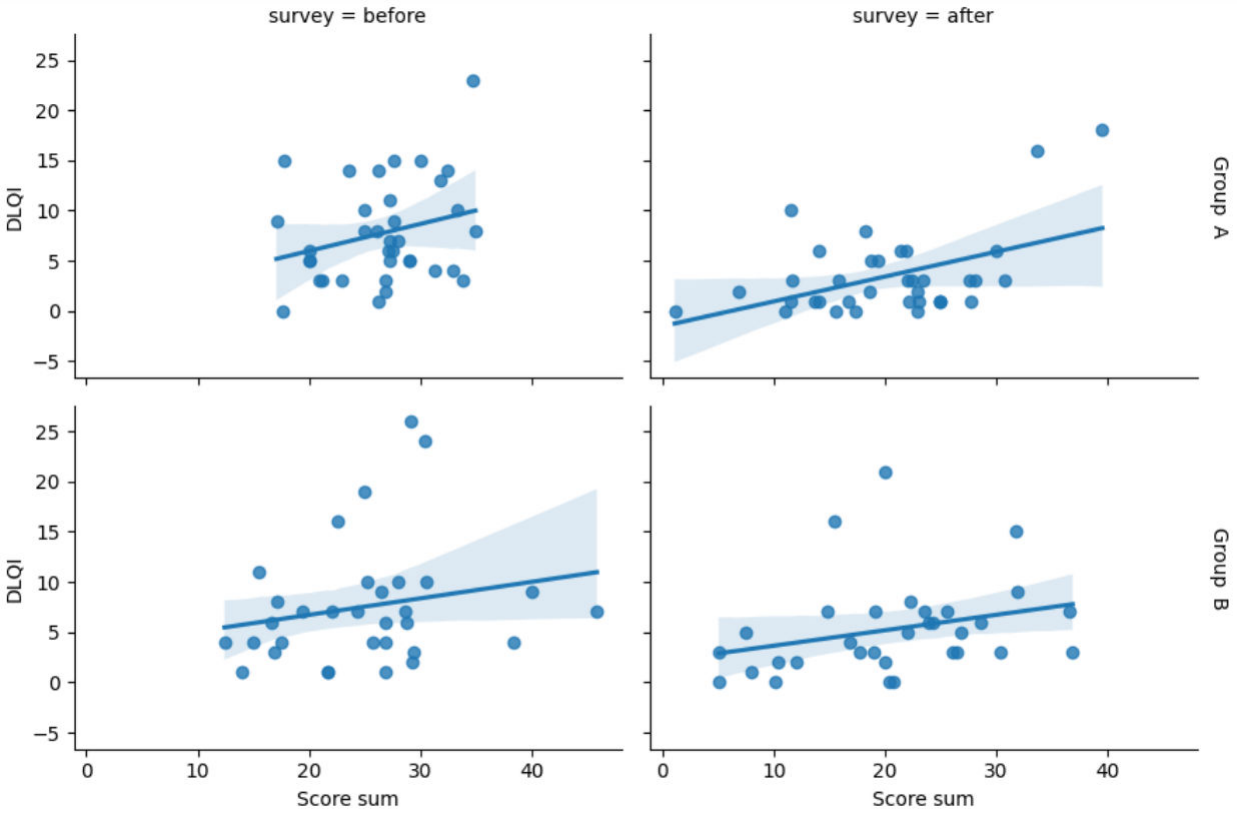
Correlation between acne severity (self-assessed) and DLQI before and after intervention in groups A and B. Abbreviations DLQI, Dermatology Life Quality Index.

Weak and moderate correlations were observed between acne severity (self-assessed) and the DLQI at baseline (*r*=0.218, 95% CI −0.12 to 0.51; *P*>.05) and after the 8-week treatment (*r*=0.279, 95% CI −0.05 to 0.56; *P*>.05) in group A, respectively. In group B, a weak correlation was observed between acne severity and the DLQI at baseline (*r*=0.244, 95% CI −0.11 to 0.55; *P*>.05) and at the 8-week follow-up (*r*=0.388, 95% CI 0.05-0.65; *P*=.03; Figure 4).

### Adverse Reactions

Overall, 3 patients in group B reported minor adverse events in the form of blushing, stinging, and itchy sensations upon the first application on day 1. They contacted NØIE and were instructed to discontinue the products and find alternative products the same day. Notably, the alternative products were well tolerated. However, none of the patients in group A reported any adverse events.

### Changes in Hypothetical Medical Intervention From the Blinded Dermatologists

The mean (SD) severity score in group A at baseline was 2.97 (1.77), which improved to 2.22 (1.66) after 8 weeks of intervention (mean difference 0.75 [1.84]; *P*=.05). After the 8-week intervention, 42.9% of the patients (14/32) were administered only dermo-cosmetics. Multimedia Appendix 2 illustrates the need for a hypothetical medically prescribed treatment for AV at baseline and after the 8-week follow-up for each intervention.

## Discussion

Precision medicine is an emerging approach in the field of dermatology, and recent clinical research has shown that the impact and management of skin diseases differ among patients. This has resulted in an increased focus on the development of personalized treatment approaches to optimize treatment response, minimize adverse reactions, and improve the overall QoL of patients [11,12]. However, the knowledge of optimal personalized treatment approaches in clinical practice remains limited.

### Main Findings

This 8-week, evaluator-blinded, randomized controlled trial, based on patient-taken photographs, evaluated the effectiveness of a novel machine learning approach for predicting the optimal treatment for mild-to-moderate AV, using dermatological evaluation combined with self-assessment. We found that IGA scores, as assessed by board-certified dermatologists, demonstrated a significant reduction in acne severity in group A compared with that in group B, indicating that personalized product recommendations generated by a machine learning model were more effective in improving acne severity than a self-selected treatment approach.

Furthermore, a significant improvement in acne severity (self-assessed) was observed after the 8-week treatment in both groups. Significant reductions in acne symptoms, such as blackheads, whiteheads, pimples, and skin oiliness, were observed in group A compared with that in group B. Therefore, this finding adds to the potential benefits of personalized treatment approaches for patients with AV.

### Interpretation

Previous studies have reported that even mild acne significantly impacts the psychological well-being and QoL of patients [12,13]. In our study, a significant improvement was observed in the QoL of patients in group A after the 8-week treatment but not in group B. This suggests that the improvement in QoL was greater with personalized product recommendations than with self-selected products. Furthermore, a weak correlation was found between acne severity and the DLQI in both groups, suggesting that the impact of acne on QoL is weakly correlated with its severity, which highlights the complexity of clinical assessments and the psychological impact of acne. These findings align with those of previous reports showing no correlation between acne severity and QoL impairment [14,15].

Minor adverse reactions, including blushing, stinging, and itchiness, alone were reported from the use of self-selected products. However, no minor or severe adverse reactions were reported from the use of customized treatment. Therefore, this finding indicates that personalized treatments may have a better safety profile than self-selected products.

Medication should ideally be the last resort in treatment given the potential side effects, intolerance, and restrictions on long-term use. Consequently, treatment approaches should prioritize resolving conditions through the least invasive methods when possible. For mild-to-moderate AV, dermo-cosmetic treatments should be considered the first line of treatment, emphasizing gentler options before resorting to medication.

### Strengths and Limitations

The strengths of this study include its randomized design which ensured the assigning of unbiased treatment and the relatively large group of patients. Furthermore, two board-certified dermatologists assessed disease severity using the IGA score. A substantial agreement was noted between the IGA assessments of the two board-certified dermatologists regarding the dynamics of acne evolution throughout the trial. However, IGA scores are limited to the face and do not cover disease activity on the back or chest. Changes in the IGA score are small, making it difficult to be used in clinical settings with interventions [5]. The Global Acne Grading System, another score that might be more suitable for research purposes, is a broader scoring system and provides a more detailed picture of disease severity; however, it is time-consuming and difficult to use in clinics with limited time [16]. This study’s limitations include a short follow-up time (8 week), a primary focus on mild-to-moderate AV, and a self-assessment of acne severity. Furthermore, self-assessment methods have been reported to be unreliable because it is difficult for patients to objectively score the severity of their acne [17]. Further research is needed in personalized skincare and machine learning models with longer follow-up times.

### Conclusions

The use of a machine learning model for personalized skincare recommendations significantly improved the severity of acne, reduced symptoms, and improved the overall QoL of patients with mild-to-moderate AV. Therefore, these findings support the potential of machine learning-based personalized treatment options in dermatology.

## Supplementary material

10.2196/60883 Multimedia Appendix 1 Supplementary Table 1. Overview of the six interventions.

10.2196/60883 Multimedia Appendix 2 Supplementary Figure 1. Need for medically prescribed interventions.

10.2196/60883 Checklist 1 CONSORT 2010 checklist

## Abbreviations

AV: acne vulgaris
CONSORT: Consolidated Standards of Reporting Trials
DLQI: Dermatology Life Quality Index
IGA: Investigator Global Assessment scores
QoL: quality of life
