# Supplementary material for: Effectiveness of a Machine Learning-Enabled Skincare Recommendation for Mild-to-Moderate Acne Vulgaris: 8-Week Evaluator-Blinded Randomized Controlled Trial
Source: JMIR Dermatol. 2025 Jul 16;8:e60883. doi: 10.2196/60883 (PMC12310563; doi:10.2196/60883)
Supplement: Multimedia Appendix 1 [file derma-v8-e60883-s001.docx]

### **Supporting information – Table 1**

Supporting Table 1: Overview of the six interventions.

| Intervention # | Products/APIs prescribed |
| --- | --- |
| 1 | Dermo-cosmetics |
| 2 | Topical benzoyl peroxide, emollients, and sunscreen |
| 3 | Topical retinoids, emollients, and sunscreen |
| 4 | Topical retinoids and benzoyl peroxide, emollients, and sunscreen |
| 5 | Topical retinoids and antibiotics, emollients, and sunscreen |
| 6 | Systemic antibiotics, topical retinoids and benzoyl peroxide, emollients, and sunscreen |

Abbreviation: API: Active Pharmaceutical Ingrediens.
