# Supplementary material for: Effectiveness of a Machine Learning-Enabled Skincare Recommendation for Mild-to-Moderate Acne Vulgaris: 8-Week Evaluator-Blinded Randomized Controlled Trial
Source: JMIR Dermatol. 2025 Jul 16;8:e60883. doi: 10.2196/60883 (PMC12310563; doi:10.2196/60883)

### **Supporting information – Figure 1**

Supporting Figure 1: Need for medically prescribed interventions.


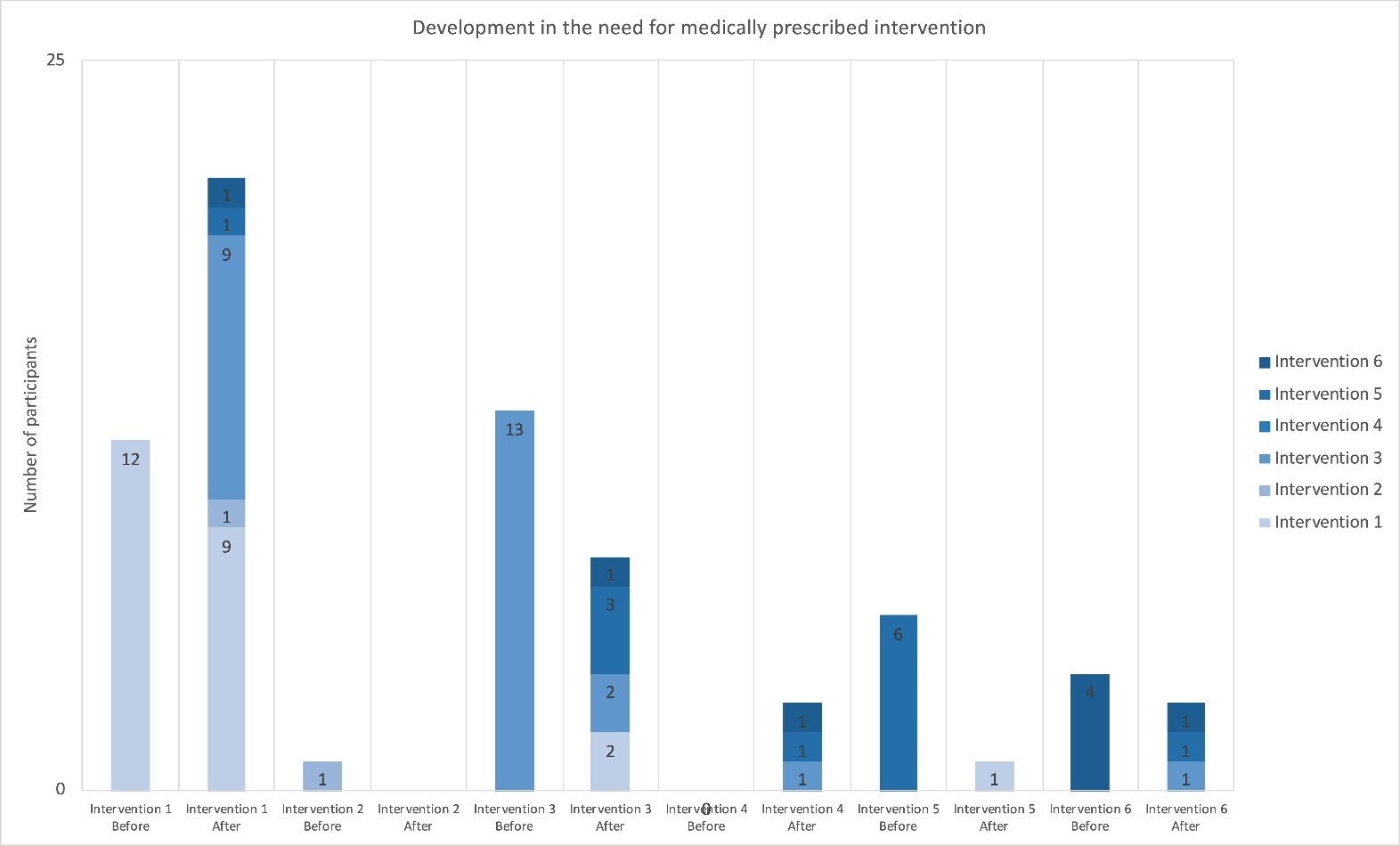

Supplement: Multimedia Appendix 2 [file derma-v8-e60883-s002.docx]
